# Totally implantable catheter embolism: two related cases

**DOI:** 10.1590/S1516-31802008000600011

**Published:** 2008-11-06

**Authors:** Rodrigo Chaves Ribeiro, Áurea Cristina Ferreira Monteiro, Quirino Cavalcante Menezes, Sérgio Tomaz Schettini, Sonia Maria Rossi Vianna

**Keywords:** Catheters, indwelling, Catheterization, central venous, Embolism, Catheterization, Antineoplastic combined chemotherapy protocols, Cateteres de demora, Cateterismo venoso central, Embolia, Cateterismo, Protocolos de quimioterapia combinada antineoplásica

## Abstract

**Context AND OBJECTIVE:**

Long-term totally implantable catheters (e.g. Port-a-Cath^®^) are frequently used for long-term venous access in children with cancer. The use of this type of catheter is associated with complications such as infection, extrusion, extravasation and thrombosis. Embolism of catheter fragments is a rare complication, but has potential for morbidity. The aim here was to report on two cases in which embolism of fragments of a long-term totally implantable catheter occurred.

**DESIGN AND SETTING:**

Case series study at Hospital do Servidor Público Estadual, São Paulo.

**METHODS:**

Retrospective review of catheter embolism in oncological pediatric patients with long-term totally implantable catheters.

**RESULTS:**

The first patient was a 3-year-old girl diagnosed with stage IV Wilms’ tumor. Treatment was started with the introduction of a totally implantable catheter through the subclavian vein. At the time of removal, it was realized that the catheter had fractured inside the heart. An endovascular procedure was necessary to remove the fragment. The second case was a boy diagnosed with stage II Wilms’ tumor at the age of two years. At the time of removal, it was noticed that the catheter had disconnected from the reservoir and an endovascular procedure was also necessary to remove the embolized catheter.

**CONCLUSION:**

Embolism of fragments of totally implantable catheters is a rare complication that needs to be recognized even in asymptomatic patients.

## RESUMO

**Contexto E OBJETIVO:**

Os cateteres de longa permanência totalmente implantáveis (por exemplo, Port-a-Cath^®^) são muito utilizados para acesso venoso de longa duração em crianças com câncer. O uso deste tipo de cateter está associado a algumas complicações como infecção, extrusão, extravasamento e trombose. A embolia de fragmento de cateter é complicação rara, mas com potencial morbidade.º objetivo do estudo foi relatar dois casos em que houve embolia de fragmento de cateter de longa permanência.

**TIPO DE ESTUDO E LOCAL:**

Série de casos do Hospital do Servidor Público estadual, São Paulo.

**MÉTODOS:**

Estudo retrospectivo das embolias de cateter de longa permanência em crianças.

**RESULTADOS:**

No primeiro caso, descreve-se uma menina com diagnóstico de tumor de Wilms estádio IV aos três anos de idade. Iniciou tratamento com implante de cateter de longa permanência em veia subclávia. Três anos após diagnóstico, foi programada a retirada do cateter por término de tratamento. No momento da retirada, houve fratura intracardíaca do cateter. Houve necessidade de retirada do fragmento intracardíaco com hemodinâmica. O segundo caso tratava-se de um menino com diagnóstico de tumor de Wilms estádio II aos dois anos de idade. Teve o cateter implantado no início do tratamento. No momento da retirada por fim de tratamento, o cateter já havia desconectado e embolizado. O fragmento intracardíaco foi retirado também por procedimento de hemodinâmica.

**CONCLUSÃO:**

A embolia de fragmentos de cateter em cateteres de longa permanência é uma complicação rara, mas que necessita ser reconhecida, mesmo em pacientes assintomáticos.

## INTRODUCTION

Venous access is extremely important in children with cancer. It is used for infusing medications, chemotherapy agents and parenteral nutrition and for collecting test samples. Moreover, some chemotherapy agents have sclerosing action in peripheral vessels. Thus, long-term totally implantable catheters (e.g. Port-a-Cath^®^) are greatly used. Today, the use of these catheters is standard for children undergoing several chemotherapy cycles.

However, this kind of catheter is associated with some well-known complications such as infection, extrusion, extravasation and thrombosis.^1,2^ Embolism of catheter fragments is a rare complication^3^ but, if it occurs, an invasive procedure for removing the catheter fragment will be needed.

## OBJECTIVE

The aim of this study was to report on two cases of fracturing and embolism of a long-term catheter at our center, within a two-year period.

## METHODS

This was a retrospective review of oncological patients with long-term totally implantable catheters at Hospital do Servidor Público de São Paulo, over a two-year period. The files of patients with catheter embolism were reviewed.

## RESULTS

Two patients had catheter embolism during this period.

**Figure 1:**
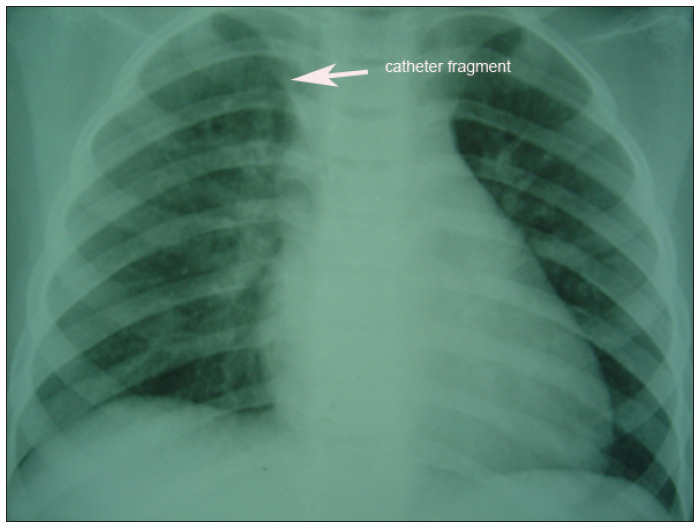
Chest radiography showing a catheter fragment in the right ventricle.

**Case 1.** L.P., a girl diagnosed with Wilms’ tumor (stage IV: presence of pulmonary metastasis) at the age of three years, underwent a chemotherapy program based on the International Society of Pediatric Oncology (SIOP) protocol. A Port-a-Cath^®^ was implanted through the right subclavian vein at the beginning of the treatment. No catheter-related complications occurred. Three years after implantation, during the removal procedure, there was unusual resistance to its retrieval. The distal portion fractured and embolized. Chest radiography showed this fragment to be in the right ventricle (Figure 1). It was removed using an interventional endovascular procedure, by means of a Seldinger puncture in the right femoral vein.

**Figure 2:**
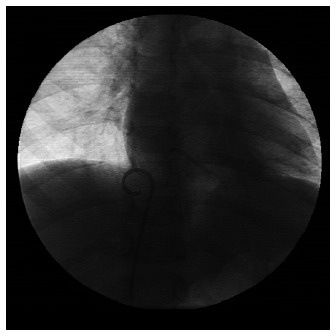
Interventional endovascular procedure to retrieve catheter fragment.

**Case 2.** N.O.F., a boy diagnosed with stage II Wilms’ tumor at the age of two years, underwent a chemotherapy program based on the SIOP protocol. A Port-a-Cath^®^ (5.4 F low-profile catheter, Arrow International) was implanted two months after the beginning of treatment. The catheter was placed through the right subclavian vein, and the reservoir was placed in the right infraclavicular region. It was removed at the end of the treatment, one year and nine months after implantation. At the time of the catheter removal, it was observed that it had fractured at the junction with the reservoir and that embolization had occurred. Chest radiography revealed that the catheter fragment was in the right ventricle. It was removed using an interventional endovascular procedure (Figure 2).

## DISCUSSION

The use of long-term catheters has been associated with various types of complications. There are complications relating to the implantation, such as pneumothorax and hemothorax, early complications that occur immediately after the implantation and late complications relating to the use of the catheter. Among the late complications, the most frequent ones are infection, extravasation, obstruction, thrombosis and extrusion.^1,2^

Embolism is a rare complication,^3^ but it has the potential for morbidity. The catheter has the tendency to embolize to the right cardiac chambers, and may reach the pulmonary artery or its branches. An interventional endovenous procedure is required in order to remove the embolized catheter fragment. Catheter fracturing and embolism can be diagnosed in asymptomatic patients by means of routine chest radiography, which reveals the position of the embolized fragment. This complication may be suspected because of problems with infusion, absence of blood reflux, extravasation and pain or edema around the reservoir during infusion. In some patients, this complication may be diagnosed during the catheter removal procedure, when fracturing and absence of a segment are found. Diagnosing the embolism and locating the embolized fragment can be done by using simple physical examination, chest radiography, radioscopy, echocardiography or Doppler ultrasound.

The mechanism for the fracturing and embolism of the catheter involves the loss of tensile strength in the catheter due to its prolonged use, and this has already been confirmed by means of physical tests and electron microscopy.^3^ Chronic compression of the catheter between the first rib and the clavicle has also been described as the cause of catheter fracturing ("pinch-off syndrome").^4^

Removal of the embolized catheter fragment is recommended, because of the risk of severe and even fatal morbidity. Nonetheless, some authors have not recommended removal in the case of fragments that present firm adhesion to the vessel wall, which occurs particularly with polyurethane catheters.^5^

## CONCLUSION

Catheter fragment embolism from totally implantable catheters is a rare complication that needs to be recognized, even in asymptomatic patients.
